# Regulating the Right to Adequate Food in Mexico: Informant Insights About the General Law on Adequate and Sustainable Food

**DOI:** 10.34172/ijhpm.9144

**Published:** 2026-07-15

**Authors:** Ashlyn Anderson, Busiso Moyo, Evelyn Núñez-González, Angela Carriedo, Jody Harris, Helen Walls

**Affiliations:** ^1^London School of Hygiene and Tropical Medicine, London, UK.; ^2^Institute of Social Development, University of the Western Cape, Cape Town, South Africa.; ^3^Institute of Development Studies, University of Sussex, Brighton, UK.; ^4^World Public Health Nutrition Association, London, UK.; ^5^Food Equity Centre, Institute of Development Studies, Brighton, UK.; ^6^Faculty of Public Health and Policy, London School of Hygiene and Tropical Medicine, London, UK.

**Keywords:** Right to Adequate Food, Food Policy, Food System, Mexico

## Abstract

**Background:**

The right to adequate food (RtAF) is increasingly incorporated into domestic food and nutrition legal frameworks to inform policy-making. Mexico’s General Law on Adequate and Sustainable Food (LGAAS), enacted in April 2024, is the country’s first law to operationalize the RtAF, which has been recognized in Article 4 of the Mexican Constitution since 2011, within domestic food policy. We examined informant insights on the LGAAS, focusing on its policy formulation and informant perspectives on the RtAF in the law.

**Methods:**

We conducted a review of legislative documents and semi-structured key informant interviews. The document review covered the period from the law’s introduction to parliament (2020) to its approval (2024). Interviews (13) were conducted with informants from the Mexican government, academia, international development partners, and civil society involved in or influencing the formulation of the LGAAS. The health policy triangle framework informed the interview guide development, while both deductive and inductive thematic approaches guided the analysis.

**Results:**

Informants identified how the following key factors affected the formulation of the LGAAS: regional action influenced RtAF legislation, national intersectoral collaboration fostered accountability, coalition building sustained momentum, and an enabling policy environment supported prioritization of the LGAAS. Political contentions and perceived industry influence also impacted negotiations. Informants consistently framed the RtAF as a cross-cutting concept linked to food security and food sovereignty, but there were differing interpretations of the state’s role in the progressive realization of the RtAF to deliver food system transformation in Mexico.

**Conclusion:**

The LGAAS positions Mexico among a small number of countries adopting a rights-based framework to address food system reform. Its formulation highlights the role of several key factors in supporting the operationalization of the RtAF into an actionable framework law, though implementation is yet to occur.

## Background

###  Origins of the Right to Adequate Food

**Key Messages**

**Implications for policy makers**

- The process of formulating the General Law on Adequate and Sustainable Food (LGAAS) was enabled by coalitions of actors from government agencies, civil society, academia, and international development partners with a shared commitment to the right to adequate food (RtAF).
- Addressing contentions in the formulation of the LGAAS required acknowledging and defining the role of the state in delivering on responsibilities to respect, protect, and fulfill the RtAF.
- Key informants viewed the RtAF as a cross-cutting tool bringing together sectors such as agriculture, environment, and health, demonstrating a holistic approach to transforming the national food system.
- The operationalization of the RtAF into framework law designed to regulate the RtAF in Mexico can provide insight for the formulation of similar framework laws in other national contexts, especially in the Latin America and the Caribbean (LAC) region.

**Implications for the public**

In Mexico, the General Law on Adequate and Sustainable Food (LGAAS) was the first piece of legislation designed to regulate the right to adequate food (RtAF), which has been recognized in the constitution since 2011. As global attention to rights-based approaches in food and nutrition policy-making increases, Mexico’s formulation and approval of the LGAAS offers an important case for examining how key informants conceptualize the RtAF and its role in policy formulation. This study’s focus on key factors shaping the LGAAS formulation – examined through the perspectives of informants – contributes empirical depth to debates about the practical value of rights-based approaches in food policy. This can help to identify opportunities to support effective implementation of the LGAAS moving forward.

The right to food was first recognized in 1948 in the Universal Declaration of Human Rights as a core component of an adequate standard of living. This recognition was later codified in the 1966 International Covenant on Economic, Social and Cultural Rights (ICESCR), which enshrined the right to adequate food (RtAF). The scope and content of the RtAF were further elaborated in General Comment 12 (1999), which clarified its key dimensions, including availability, accessibility, adequacy, and sustainability of food for all people.^1,2^

 Under international human rights law, the state is the primary duty bearer, with obligations to respect, protect, and undertake actions towards the progressive fulfilment of the RtAF.^3,4^ Achieving this progressive realization requires ensuring food security – such that all people at all times have physical, social and economic access to sufficient, safe and nutritious food that meets their dietary energy requirements and food preferences.^5,6^ Considerations of nutrition are therefore integral to the adequacy component of the RtAF, as adequacy requires that food meet both a person’s nutritional requirements and be safe for health.^7^

 Legal developments related to the RtAF have strengthened its political salience, underscoring the importance of documenting and critically examining such developments where they occur.^8-10^ This study contributes to this literature by examining the example of legislative advances on the RtAF in Mexico, specifically through the perspective of key informants involved in the policy-making process.

###  Operationalization of the Right to Adequate Food

 The RtAF, as articulated in international law, provides the normative foundation for legal instruments that can be operationalized at the national level through state action. Over recent decades, an increasing number of countries have adopted explicit measures to protect the right to food, either by incorporating it in their constitutions, or by enacting rights-based food security legislation, strategies and policies.^10-12^ In 2024, 42 countries had explicitly or implicitly enshrined the RtAF in their constitutions, albeit with varying degrees of legal recognition and enforceability.^13^

 Beyond constitutional provisions, governments may give effect to the RtAF through legislation, including through the adoption of framework laws as instruments for implementing national strategies.^14^ Framework laws establish a general legal basis in a specific policy domain through articulating overarching principles and obligations, while leaving detailed policy measures and implementation mechanisms to sector-specific legislation.^14^ Such laws are especially important for strengthening accountability for the RtAF in contexts characterized by persistent malnutrition and rapidly changing food environments.^15^

###  The Right to Adequate Food in Latin America and the Caribbean 

 The Latin America and the Caribbean (LAC) region has made substantive developments in developing legal frameworks that promote awareness, protection and justiciability of the right to food. In Latin America, legal obligations of states related to the RtAF derive from several international treaties and regional declarations, including the Hunger-Free Latin America and Caribbean Initiative 2025.^16,17^ The Hunger-Free Latin America and Caribbean Initiative has fostered regional collaboration and informed efforts to promote constitutional recognition of the RtAF, as well as rights-based approaches to food and nutrition policy-making across the LAC region.^17^

 Previous research has documented the regional movement in legislation on the RtAF, characterized by two distinct waves.^16^ The first wave, from 2005-2009, resulted in the adoption of six national and ten sub-national laws recognizing the RtAF. The second wave focused on advancing pending legislative drafts and implementing the institutional frameworks created by the laws.^16^ The LAC region currently includes 16 countries with explicit constitutional provisions recognizing the RtAF as a guide for food and nutrition policy-making.^14,16^

 Despite these advances, significant variability in political commitment and parliamentary action, and action of specific stakeholder groups across countries highlights the need for country-specific analyses. Such analyses are essential for understanding the formulation and implementation of laws designed to regulate the RtAF, as well as how the RtAF is perceived by key actors.

###  Mexico’s Legislative Process and Recognition of the Right to Adequate Food 

 Within the national legislative context, Mexico’s parliamentary recognition of the RtAF has evolved over time. A significant milestone occurred in 1981, when Mexico became a state party to the ICESCR, thereby committing to work towards the progressive realization of access to nutritious, sufficient, and quality food for all.^17-19^ Over subsequent decades, economic liberalization associated with the North American Free Trade Agreement and politically conservative administrations reshaped the relationship between the state and the private sector, expanding market openness and industry influence over public policy.^18^ During this period, human rights, including those related to food, had not been fully operationalized into legal instruments. This changed in 2011, when advocacy from civil society and the political left led to the inclusion of human rights in public policy domains such as education, health and food.^18,20,21^ That year, the Chamber of Deputies approved a constitutional reform establishing the RtAF in Article 4, which states “*Every person has the right to nutritious, sufficient, and quality food. The State will guarantee it.”*^18,19^

 Since the 2011 constitutional reform, Mexico has adopted 175 food- and nutrition-related policies that explicitly reference the right to food.^21^ However, it was not until 2024 that a comprehensive framework law was developed to structure and guide the regulation of the RtAF in a more integrated, cross-sectoral manner. On April 18, 2024, Mexico approved the General Law on Adequate and Sustainable Food (*Ley General de Alimentación Adecuada y Sostenible*, LGAAS) as a framework law grounded in the RtAF, designed to modify and complement existing national food policy.^18,19^ The LGAAS mandates national policy measures to prevent malnutrition and food insecurity, explicitly recognizing these as interconnected issues that can be addressed throughfood systems transformation, including changes to food production, supply chains, distribution, and food environments.^22^

 Mexico’s regulatory framework includes the Mexican constitution at its apex, under which general laws are enacted. These general laws are further operationalized through regulations, agreements, and guidelines, each with different degrees of reach and scope. In the specific case of the LGAAS, the legislative process followed standard congressional procedures: the draft law was first approved by the Senate, and subsequently by the Deputy chamber, with approval by both chambers required for enactment. Once passed by Congress, the bill was submitted to the President, who could either sign it into law or exercise a veto. Primary legislation is drafted and approved by the federal government, specifically the Mexican Congress, and is published in the Diario Oficial de la Federacion. Once the laws are approved (either proposed by the legislative or executive branch), the National Commission for Regulatory Improvement coordinates regulatory policy across all levels of government, supporting the development of secondaryregulations, technical standards, and administrative agreements that operationalize the general law.^18^

 Since the approval of the LGAAS in 2024, awareness has grown regarding contemporary policy developments on the RtAF and the role of governance processes and coalition-building.^10-12^ A small number of studies specifically document the LGAAS as one of the most ambitious attempts to operationalize the RtAF.^11,19,22^ Yet there is a limited body of empirical research examining how RtAF commitments are translated into domestic policy formulation processes in the context of framework legislation, specifically through key informant insights. Addressing this gap is important because the realization of the RtAF through the LGAAS depends not only on legal recognition, but on how rights are negotiated, prioritized, and institutionalized during policy formulation. Empirical analysis of these processes contributes to RtAF scholarship beyond normative theory and legal doctrine toward a deeper understanding of the political dynamics that impact rights-based food policy.

 The aim of this study was to examine informant insights on the LGAAS, focusing on its policy formulation and informant perspectives on the RtAF in the law. Thus, this study provides critical insight into how key informants perceive how the RtAF can shape policy agendas, alliances, and implementation expectations, thereby contributing empirical depth to debates about the practical value of rights-based approaches in food policy.

 This research is grounded in the understanding that the policy formulation process is not a single or linear decision point but an iterative process involving formal and informal dynamics.^23^ These dynamics include the various actors involved; the broader context in which human rights, particularly the RtAF, are interpreted and understood by policy-makers; the advocacy strategies used to advance the law; and the negotiation and drafting processes that ultimately shape the final policy text.^24-27^ Specifically, this study seeks to examine the informant insights on the “bit in the middle” of policy formulation, which refers to the crucial often overlooked stage between setting a policy agenda and its implementation.^23^

## Methods

 This qualitative study draws primarily on key-informant interviews, complemented by a review of publicly-available documents from when the law was presented to parliament to its approval in the legislative process.

###  Data Collection 

 We conducted semi-structured interviews with 13 key informants. Based on publicly available records (eg, congressional records, legislative committee documents, and policy reports), and a review of publicly available legislative documents, participants were identified from across public health, government, academia, an international development organization, and civil society as actors who had directly contributed to or influenced the legislative process. Purposive sampling was used to identify individuals with relevant expertise and involvement in the formulation of the LGAAS. Following purposive sampling, we utilized a snowball sampling approach through professional networks to identify additional potential participants, aiming to capture a diversity of perspectives and strengthen the validity of the findings. We attempted to recruit government informants and legislators in senior roles, but this was not possible due to time constraints and informant availability, and our interviewees are from a range of levels of seniority across government ministries.

 All interview documents and transcripts, including the interview guide, were originally drafted in Spanish and reviewed by bilingual co-authors (AA, supported by AC and ENG) all with extensive experience in Mexico. All of the semi-structured interviews were conducted with key informants in Spanish (AA).

 A total of 24 invitation emails accompanied by a participant information sheet and informed consent form were sent in Spanish. All documents received internal institutional ethical review approval prior to contacting potential participants. Invitees were offered the option to conduct the interview in either Spanish or English. Two individuals declined to participate and nine did not respond. A total of 13 interviews were ultimately conducted, and after this point no additional interviews were pursued as theoretical data saturation had been reached and no new themes were discussed.^28^ The final sample (Table 1) included representatives from government, academia, civil society, and an international development organization, with knowledge of the LGAAS and/or the RtAF in Mexico. Government informants, including representatives from government ministries and national research institutes, were the most represented.

**Table 1:** Actor Type and Sector of Informants Interviewed

**Sector **	**Organization **	**Number of Interviewees**
Government	Ministry of Agriculture and Rural Development	5
	Ministry of Health	
	National Institute of Public Health	
Academia	Instituto Tecnológico y de Estudios Superiores de Monterrey [Technological Institute of Higher Education of Monterrey]	2
	IDS	
Civil society	El Poder de Consumidor [The Power of the Consumer]	4
	ANEC	
	The Hunger Project Mexico	
International development organization	FAO, LAC region	2

Abbreviations: IDS, Institute of Development Studies; ANEC, Asociación Nacional de Empresas Comercializadores de Productores del Campo [National Association of Marketing Companies of Rural Producers]; FAO, Food and Agriculture Organization of the United Nations; LAC, Latin American and the Caribbean.

 All interviews were conducted online between July and August 2024, in Spanish, and followed a semi-structured interview guide. Informed consent was obtained from all participants prior to each interview. The interview transcripts were labelled with informant identification numbers and securely stored in an institutional OneDrive to ensure confidentiality.

 The development of the interview guide to examine informant insights on the LGAAS, focusing on its policy formulation and informant perspectives on the RtAF in the law, was guided by the health policy triangle developed by Walt and Gilson.^24^ This framework examines key components—policy content, context, actors, and process—shaping health policy processes.^24^ The health policy triangle has been widely used in the literature to analyze legal and policy developments across diverse health-related topics, and in relation to different aspects of the policy cycle.^25^

 Interviews in Spanish lasted approximately 60 minutes and were conducted using a secure institutional Microsoft Teams account. Detailed notes were taken in Spanish during each interview. Immediately following each interview, recordings were reviewed to facilitate immersion in the data and identify initial themes. Interviews were transcribed and subsequently translated using TurboScribe software into English for analysis.

 To contextualize the informant responses on the timeline of the LGAAS formulation, a review of key documents was conducted to provide an overview of the legislative process leading to the law’s approval. The review also led to the development of pre-determined codes, which were used in the coding process of the interviews (Table 2). The documents reviewed included the final text of the law, draft initiatives, first reading reports, public statements, reservations to specific articles, successive drafts of the LGAAS, and legislative reports (Table 3). All documents were originally produced in Spanish and translated to English for analytical purposes.

**Table 2:** Predetermined Codes

**Secondary Level Code**
Primary objective of the LGAAS
Role of the RtAF
Situational factors
Structural factors
International factors
Cultural factors
Problem identification
Agenda setting
Law formulation process
Enabling factors
Constraining factors
Role of coalitions
Role of civil society
Role of government
Role of industry
Role of international actors/coalitions

Abbreviations: LGAAS, General Law on Adequate and Sustainable Food; RtAF, right to adequate food.

**Table 3:** Legislative Documents of the General Law on Adequate and Sustainable Food Reviewed

**Title **	**Type of Document **	**Authors**	**Date of Publication **
Initiative that issues the General Law of the Right to Adequate Food	Draft decree	Deputy Diego Eduardo Del Bosque Villarreal	October 2020
Initiative to enact the General Law on Adequate Food	Draft decree	Senator Ana Lilia Rivera Rivera, referred to the Joint Committees on Social Development and Welfare, and on Legislative Studies, Second Committee	November 3, 2021
Draft decree initiative to enact the General Law on Sustainable Food	Draft decree	Senator Nancy de la Sierra Arámburo, referred to the Joint Committees on Social Development and Welfare, and on Legislative Studies, Second Committee	December 7, 2021
Reservation to the opinion of the Joint Committees on Social Development and Welfare regarding the draft decree by which the General Law on Adequate and Sustainable Food is issued	Reservation to Article 22, 21, and 1 in the LGAAS	Senator Claudia Ruiz, Massieu Salinas	April 27, 2023
Reservation to the opinion of the Joint Committees On Social Development and Welfare and on legislative studies regarding the draft decree to issue the General Law on Adequate and Sustainable Food	Reservation to Article 21 in the LGAAS	Senator Noé Fernando, Castañón Ramírez	April 27, 2023
Report containing the draft of the General Law on Adequate and Sustainable Food	First reading report	From the Joint Committees on Social Development and Welfare; and on Legislative Studies, Second Committee	April 28, 2023
Final report containing the draft of the General Law on Adequate and Sustainable Food	Legislative report document	Senate Committee	September 19, 2023
Contributions to the parliamentary debate	Legislative debate document	Concepción Torres Ramírez, Institute of Belisario Domínguez, Senate of the Republic	October 2023
General Law on Adequate and Sustainable Food	Legislative document	Coalition of Senators and Legislators from the Chamber of Deputies and Senate	April 18, 2024
Exhort in support of the Initiative of the General Law on Adequate and Sustainable Food, addressed to the Senate of the Republic	Debate document	Coalition of civil society, social movement, institutions, and international organizations	April 18, 2024

Abbreviation: LGAAS, General Law on Adequate and Sustainable Food.

 The legislative document review involved identifying publicly available documents through searches of government websites of the Ministry of Agriculture and Rural Development, from where the law originated, as well as the Mexican Chamber of Deputies and Senate, and official government publications such as the Official Gazette. Inclusion criteria required that documents focus explicitly on the LGAAS or on draft versions of the law prior to its approval. Key search terms were applied in both Spanish and English, including: “*general law for adequate and sustainable food*,” “*right to adequate food,*” and “*Mexico*.” The search was restricted to the period from 2020, when the LGAAS was first presented in parliament, to 2024, when the law was approved. The review was limited to publicly available materials directly related to the LGAAS. In total, ten legislative and policy documents met the inclusion criteria and were reviewed. These documents were primarily used for cross-analysis with the interview data, enabling corroboration of timelines, processes, and key developments.

###  Data Analysis and Interpretation 

 The interview data were analyzed using NVivo10 software through a combination of deductive and inductive thematic approaches. Translations of interview transcripts and documentary materials into English were undertaken after initial familiarization with the data in Spanish and were reviewed iteratively by bilingual co-authors (AA, AC, and ENG) to preserve nuance, terminology, and context-specific meanings. Where concepts or terms were closely tied to the Mexican legal or policy context, these were discussed among the bilingual co-authors during analysis to ensure accurate interpretation rather than relying on more literal translations. Through iterative analysis of the informant interviews, inductive codes were developed based on recurring themes and patterns in the data, which were subsequently compared and integrated with the predetermined codes, based on the health policy triangle framework (Table 2).

 The information from the legislative document review was analyzed with key informant interview data to provide deeper insights into informants’ beliefs, ideas, concepts, and narratives relevant to the study objectives.^29^ The collection of both documentary and interview data enabled the capture of both formal and informal dimensions of the LGAAS formulation process, contributing to a more nuanced understanding of the law’s formulation.^30^ This combined approach enabled a structured analysis while also accommodating the identification of novel themes and insights from the data.

## Results

###  Informant Insights on the Formulation of the LGAAS

####  Regional Action Influenced the Right to Adequate Food Legislation 

 The initial stages of formulation of the LGAAS in Mexico were described as being strongly influenced by legislative advances on the RtAF across the LAC region. Two informants from civil society organizations highlighted Mexico’s role as a regional leader in advancing food governance and nutrition policy, which helped pave the way for the LGAAS. A government informant noted that regional experience with framework laws on the RtAF created receptivity among policy-makers in Mexico.

 “*We were no longer proposing a law with unknown results, but we had evidence of it in other countries with more or less similar circumstances”* (Government Informant, Interview #11).

 Regional collaboration and coalition-building with other countries in LAC also played a key role in shaping the formulation of the LGAAS. Civil society and international development organization informants emphasized that the development and adoption of the law was facilitated by the Parliamentary Front Against Hunger in Latin America and the Caribbean (FPH-ALC in Spanish), a network of over 400 legislators representing diverse political parties. Founded in 2011, the FPH-ALC served as a platform to prioritize hunger and food insecurity on political agendas, via the Framework Law on the Right to Food and Food Sovereignty, which provided governing principles for LAC member states and informed the structure of the LGAAS.^20^ The majority of informants, including from government, civil society, and international development organizations, emphasized the essential role of the FPH-ALC as an international influence shaping both the legal framework and the conceptualization of the RtAF within the LGAAS. Additionally, informants commented that international organizations, such as the Food and Agriculture Organization of the United Nations, also influenced the initial development of the law, particularly providing technical support to ensure that nutrition and legal aspects of the RtAF remained central to the law.

####  National Intersectoral Collaboration Fostered Accountability 

 All 13 informants commented on intersectoral collaboration across sectors to translate normative commitments on the RtAF into concrete definitions, institutional arrangements, responsibilities, and priorities.

 According to government and civil society informants, the parliamentary process for developing a legal framework to regulate the RtAF with the provision to ensure access to nutritious, sufficient, high-quality, and sustainable food took four years in the Mexican Chamber of Deputies and Senate. This is confirmed by the draft rulings from the LXV Legislature which document that in October 2020, Deputy Diego del Bosque of the LXIV Legislature led efforts to introduce initiative through the Chamber of Deputies.^18^ However, the proposal lacked sufficient support from other parliamentary groups and did not advance to drafting or voting.

 The catalyst for the drafting and design of the LGAAS occurred in March 2021 when the legal formulation became a collaborative effort through the intersectoral working group Interagency Group of Health, Food Environment, and Competitiveness (GISAMAC), which included representatives from federal government, academia, and civil society organizations. Civil society organizations such as El Poder del Consumidor, Salud Crítica and the Hunger Project Mexico contributed ideas, comments and proposals that informed the drafting of the law. The majority of civil society informants commented that the LGAAS built on GISAMAC’s foundation to establish the National Interagency System of Health, Food, Environment and Competitiveness (SINSAMAC), which was embedded in Title V of the law. Headed by the President of Mexico, SINSAMAC is responsible for defining and implementing national food policy through Food Councils at federal, state, municipal and local levels, each supported by intersectoral boards.^18^

 Government informants described the creation of SINSAMAC in the LGAAS as a key step in uniting previously siloed actors in food governance and fostering collective accountability. Both government and civil society informants characterized the LGAAS as *“very ambitious”* and *“profoundly revolutionary”* because of the establishment of SINSAMAC, which takes a holistic approach to food policy and governance.

 “*SINSAMAC reorders many of the powers that the secretariats have and brings intersectoral collaboration, such that SINSAMAC begins to lower the operation of this law down to the state level. So, the successful operation of the SINSAMAC is essential to the operation of the law.” *(Civil society Informant, Interview #2).

####  Coalition-Building Sustained Momentum 

 Civil society and academic informants discussed collaborative relationships and coalitions built between policy-makers and civil society organizations to sustain the momentum and continue advocating for the RtAF. These partnerships helped build consensus and align priorities for the law’s advancement. Four civil society informants noted how coalition building with government evolved under the recent political administration.

 “*As a result of this six-year term, our relationship with the government was different and our participation within certain spaces. It allowed us to have a certain voice as a civil society, and it has not always been the same. I think a lot has to do with the political context and those who are in charge of decision-making*” (Civil society Informant, Interview #9).

 During the initial stages of agenda setting and formulation of the LGAAS, multiple advocacy coalitions played a key role, including civil society organizations working collaboratively with government institutions to address food systems issues. All civil society and academic informants cited the prominent role of civil society alongside many other organizations in collective efforts that accelerated the law’s prioritization. Informants cited an agreement made in 2023 across civil society groups representing peasant farmers, consumer interests, and public health urging the Development and Social Welfare Commissions of the Senate to uphold the constitutional commitments made in 2011.

 However, academic informants noted that not only did these coalitions help set the agenda for the law, but specific actors within government helped sustain momentum for subsequent stages of the policy cycle.

 “*I think that the fact that the law was created and later approved does not have much to do with the people who were allies in Congress, as often new legislators arrived and we started from scratch, right? So, I think that was the problem, that is, there was no one to grab with a very loud voice. That strong voice at this moment of approval was Senator Ana Lilia Rivera Rivera, who was the one who proposed this version” *(Academia Informant, Interview #7).

 Senator Ana Lilia Rivera Rivera of the Morena party was cited by eight informants as the “champion” of the LGAAS, leading negotiations in the Senate to prioritize policies protecting smallholder farmers and native maize from commercial interests.While her leadership was crucial for the law’s development and approval, civil society informants emphasized that the Senator’s success was closely linked to sustained advocacy and pressure from progressive groups pushing for the passage of the LGAAS.

####  Enabling Policy Environment Supported Prioritization of the LGAAS

 Informant interviews highlighted that the formulation of the LGAAS as a law to regulate the RtAF occurred within an overall enabling policy environment that helped to prioritize and eventually pass the law. Government and academic informants emphasized that the precedent for the LGAAS was facilitated by the formulation, approval, and implementation of social protection programs which incorporated elements of the RtAF, such as the Production for Wellbeing program initiated in 2019. As noted in legislative documents accompanying initiatives introduced by senators Ana Lilia Rivera Rivera and Nancy de la Sierra, these social protection programs ensured that food assistance programs, which include provisions related to the progressive realization of the RtAF, remained a policy priority across political administrations and national policies.

 Additionally, civil society informants highlighted three key policy milestones that they considered created a favorable environment for the development of the LGAAS: the 2019 front-of-package labelling regulation, the 2020 decree prohibiting glyphosate, and the designation in 2023 of the National Day of Maíz. Informants described these measures as pivotal in shaping the LGAAS by aligning RtAF discussions with the administration’s commitment to supporting rural farmers and agroecological approaches. Several civil society informants noted that progress on these policy priorities had previously stalled under earlier administrations, but brought momentum and urgency for food policies with elements of the RtAF. In reflecting on the years preceding the approval of the LGAAS, one civil society informant commented:

 “*The public conversation that existed around food was a little more sensitive at that time. I also believe that, particularly for the law, the entire dialogue process that was opened due to front of package labeling and the discussion of advertising in school settings helped to have this possibility of discussing the right to food in different spaces” *(Civil society Informant, Interview #3).

 Academic and civil society informants also commented that the final approved version of the LGAAS was facilitated by two distinct legislative initiatives that brought the law to the forefront of parliamentary priorities. On November 3, 2021, Senator Ana Lilia Rivera Rivera presented the* General Law on Adequate Food to the Senate of the Republic*, which led to a Parliamentary Forum on November 24, 2021. This forum, convened by drafting committees in collaboration with the FPH, solicited feedback on the initiative.

 On December 7, 2021, Senator Nancy de la Sierra introduced a complementary initiative that incorporated sustainability considerations, ultimately becoming the LGAAS. One academic informant highlighted the distinction between the two initiatives, noting that Senator Nancy de la Sierra’s draft emphasized climate change and environmental impacts, calling for more sustainable food production and consumption. Informants also emphasized that incorporating the terms “appropriate” and “sustainable” signaled a shift from narrow nutrition or food security framings toward a broader, systemic, food-systems approach to progressive realization of the RtAF.

 Throughout May and June 2022, following general project approval, technical secretariats collaborated with the FPH and various institutions to refine the draft based on stakeholder feedback. One government informant noted the importance of this stage, during which the joint commissions of Welfare and Social Development and the Senate approved the Draft Opinion on the LGAAS initiative and established technical working groups for detailed discussions on the draft decree.

####  Political Contentions and Perceived Industry Influence Impacted Negotiations

 In September 2023, the LGAAS was unanimously approved with 420 votes in favor, and was referenced by ten informants as a key moment in the policy-making process. However, despite the final approval of the law, informants reflected on the contentions that arose in the later stages and complex perception of the role of the private sector in the legislative negotiations.

 One government informant noted that the policy environment that was favorable to the law generally reflected the interests of the political majority, which limited the negotiation process:

 “*Here we received a first proposal or a second proposal on the general food law and it was from the political faction that is now in the government, which has a majority. So, I understand that because they have a majority, they approved it once and for all, but I get the impression that they didn’t take into account all the opinions and comments. I understand that they had some urgency to publish the law, perhaps due to situations right there in Congress. *(Government Informant, Interview #6).

 In the final stages of drafting and final approval of the LGAAS, the role of industry influence arose in the majority of informant interviews, with differing perspectives on the degree of influence. Many informants recognized the food industry as a significant threat to public health and nutrition policies in Mexico, citing historical attempts to interfere with food legislation to promote commercial interests. Informants highlighted tensions between private sector interests and public health objectives, which shaped negotiations and sometimes limited the scope of the RtAF within the law.

 Some academic informants argued that the food industry exerted indirect influence on the legislative level, instead operating primarily through lobbying, media campaigns, judicial interventions, and financial leverage that affected government decisions. In contrast, several civil society and government informants perceived that industry played a more direct role in influencing policy-makers, including from within government ministries. Disagreement remained on the topic of influence of industry stakeholders and ongoing debates about the state’s regulatory role. One government informant noted that opposition to the LGAAS during the approval process emerged from an individual within their own ministry, facilitated by alliances between the government and business interest groups:

 “*Despite these favorable conditions, the LGAAS encountered opposition and resistance from business groups and corporations linked to food systems. They have advocated that there be no regulation or transformation in food systems, so in the approval process, these interest groups found an ally in the Ministry of Agriculture” *(Government Informant, Interview #13).

###  Informant Perspectives of the Right to Adequate Food Legislation in the LGAAS

####  Leveraging the Right to Adequate Food as a Conceptual and Operational Tool

 The interviews highlighted informants’ perceptions of the RtAF in the LGAAS as both a motivational and operational tool linking cultural appropriateness, economic access, nutritional quality, and environmental sustainability of food. Civil society and government informants broadly agreed that there was strong motivation and momentum behind the concept of the RtAF. Civil society informants also framed the RtAF in the LGAAS as a cross-cutting concept that extends beyond nutrition to encompass biocultural wealth, gastronomic diversity and agrobiodiversity. This perspective aligns with Article 4 of the LGAAS, which situates food within its cultural context and emphasizes the importance of supporting healthy diets.

 Academic and government informants noted that as a legal instrument, the LGAAS helped make the RtAF operational by seeking to modify or add to current national food policy. However, the majority of civil society and academic informants noted that systemic shifts are needed in the national food system to progressively realize the RtAF. An academic informant noted that the RtAF should require regulating food environments to decrease availability of unhealthy foods and enhance the availability of healthy foods. Similarly, a civil society informant commented on the current structure of food production and consumption in relation to the RtAF:

 “*Ensuring adequate nutrition in the law means guaranteeing the right to food, and that involves food be produced in a sustainable way, which implies questioning the production models we have*”* (*Civil society Informant, Interview #2).

 Approximately a quarter of informants highlighted the importance of a healthy diet to the RtAF, and how in practice, the law must require nutrition strategies specific to each region, considering local dietary habits and resources. This is reflected in the LGAAS through regionally tailored normative baskets (*canastas normativas*) designed to meet local nutritional needs. Furthermore, a government informant commented that the RtAF in the law conceptually requires nutrition strategies specific to each region, considering local dietary patterns and resources.

####  Exploring the Relationship of Right to Adequate Food With Other Food-Related Concepts

 The informant interviews highlighted the relationship between the RtAF and other food-related concepts, including food security and food sovereignty, which also feature prominently in the LGAAS. The majority of civil society informants emphasized that the RtAF is closely related to food security and sovereignty, as the concepts of the RtAF, food security, and food sovereignty all require systemic changes to the food system. But overall, these civil society informants asserted that the concepts were distinct, with progressive realization of the RtAF depending on the achievement of both food security and sovereignty.

 Informants from government and civil society did not distinguish sharply between these concepts, viewing food security, food sovereignty, and the RtAF as complementary objectives that collectively work to ensure access to nourishing, culturally appropriate food. In contrast, four informants from academia and government highlighted the distinctiveness of the RtAF and pointed to tensions in how these concepts influence policy within the LGAAS, particularly regarding food access, quality, and autonomy. One government informant explained the concept of *auto-consumo *(food self-sufficiency) as an expression of food sovereignty that aligns with the goal of recognizing the RtAF, while critiquing the food security agenda as often driven by industry priorities, emphasizing availability over nutritional quality.

 “*I think they are related, but not the same, because food security for me is having access to [food], right? I’m going to be able to eat. The difference here is what are you going to be able to eat. I can have food security if I have availability of many ultra-processed foods”* (Government Informant, Interview #5).

####  Defining the Role of the State in the Responsibilities for the Right to Adequate Food

 Informants also had diverse interpretations of the state’s responsibility for the progressive realization of the RtAF as stated in the LGAAS. All 13 informants agreed that the state is the primary duty bearer for delivering on the RtAF, however, there was disagreement regarding the specific responsibilities the state must fulfil to meet these legal obligations of the LGAAS as a policy instrument to effectively deliver on the RtAF.

 A point of contention among government informants centered on whether the state’s role should prioritize market regulation to create enabling environments or involve direct intervention in food provision. One government informant emphasized the importance of enhancing people’s economic capacity to access nutritious food, rather than focusing solely on increasing food production.

 “*The role of the government is to facilitate access to food, not produce it. Our function is not to distribute food or give food to people, but to create the conditions so that people have a sufficient income and have food available in the place where each family lives”* (Government Informant, Interview #7).

 Additionally, two informants from government and one from academia noted that certain provisions in the law assign too many functions exclusively to the government, creating implementation challenges due to limited resources and institutional capacity. For example, Articles 40, 41, and 50 in the LGAAS assign the Ministry of Agriculture responsibilities such as managing State-Sponsored Granaries and establishing strategic reserves of basic grains and seeds through public procurement and storage infrastructure. Commenting on this legal responsibility in relation to the RtAF, one government informant explained:

 “*Here in the law, an obligation is established for the Government—federal, state, and municipal authorities—to purchase at least 15% of their food from small- and medium-scale producers who are producing in a healthy and sustainable way” *(Government Informant, Interview #11).

## Discussion

 This study revealed that the LGAAS was formulated through intersectoral governance to operationalize the RtAF from an article in Mexico’s Constitution to an actionable legislative framework. As of 2026, the law’s impact on the progressive realization of the RtAF remains to be seen.^27^ However, our analysis provides a number of findings relevant to its formulation, and informant perspectives on the RtAF.

 Regarding the formulation of the LGAAS, analysis of key informant perspectives identified several key factors that supported the development of the law, while some factors presented challenges throughout the dynamic, non-linear policy process. In line with Haynes and Loblay, rather than categorize these factors solely as barriers or enablers in the policy formulation, we have endeavored to capture the nuance and complexity of such factors in shaping the policy.^31^

 Informants identified factors that supported the formulation of the law including the actions of coalitions and collective advocacy from academia, civil society and development partners. Relatedly, previous studies on the formulation of food system policies have similarly noted the importance of strong policy champions in generating political will.^32^ For instance, research on regulatory policies to restrict marketing of unhealthy food and beverages to children in seven countries demonstrated that coalitions of civil society and academics helped governments overcome many technical and political challenges through providing strategic advocacy.^33^ Similarly, studies in Ethiopia suggest that regional administrative and political cultures are key to influencing the extent to which policies reflect local concerns.^34^

 However, the formulation of the law was not without challenges, particularly in the disagreements over the extent of industry influence in the legal negotiations. The issue of industry influence in the policy process reflects the considerable literature from Mexico and elsewhere documenting how corporate actors have strategically impeded public health policies—such as front-of-pack labelling policies, policies on trans fatty acids, and the sugar-sweetened beverage tax—exerting detrimental influence on policy-making at national and international levels.^34-37^ These dynamics highlight the critical need for strategic action to address actor power asymmetries and corporate strategies, given the strong evidence for how they shape policy-making. Such action is essential to safeguard public health and nutrition objectives and strengthen rights-based policy formulation.^37,38^

 Additionally, informant perspectives also illuminated how the RtAF influenced not only proposed solutions to food system and nutrition-related challenges but also the framing of those issues themselves. Informant beliefs are crucial to the policy context; shaping what counts as evidence, how policy problems are defined, approaches to public participation, and strategic engagement, ultimately shaping policy decisions and implementation.^34^ This was revealed in the tensions around the state’s responsibilities for the progressive realization of the RtAF. The differential interpretation of the state’s responsibilities may reflect the differing ideologies of key actor groups, particularly in relation to neoliberal economic policies, as previously outlined in the research from Cervantes et al on the food policy space in Mexico.^36^

 The development of the LGAAS parallels legal developments in South Africa, where constitutional commitments have guided the legal realization of socio-economic rights.^40,41^ Similarly, in 2024, Rwanda developed its fifth Strategic Plan for Agricultural Transformation, linking the RtAF to national food security through intersectoral planning processes and extensive interdepartmental discussions.^27^ Mexico’s experience highlights the critical role of advocacy and academic networks in providing the evidence, analysis, and foresight needed to support inclusive transitions. While several constraints arose during the formulation of the LGAAS, the law’s institutional and strategic foundations enabled multi-stakeholder participation and represent a major achievement for human rights activists in Mexico and across the LAC region.

###  Strengths and Limitations 

 A key limitation of the study was its reliance on publicly available legal documents, yielding ten documents, because official government documents in Mexico are accessible only through private archives. The study also did not include interviews with commercial actors, such as those in agribusiness or food processing, due to its focus on government, civil society, international development partners, and academia. Including industry perspectives could have provided valuable insights into potential implementation challenges and how commercial actors perceive the RtAF. Additionally, the interviews were conducted online rather than in person due to budget and time constraints. While in-person interviews may have fostered better rapport and deeper insights, previous studies have shown that video interviews can yield reliable information when in-person meetings are not feasible, particularly when both participants and interviewers are experienced with using video technology.^42^ Furthermore, two government informants requested the interview guide in advance, which may have influenced the depth of their responses relative to other participants. Despite these limitations, complementing the interviews with legislative document analysis strengthened the validity of the findings.

 The study contributes to understanding of how a framework law can operationalize the RtAF within the context of national food security.^11,27^ As such, it offers insights and learnings that may be applicable to other countries across the LAC region and beyond. Mexico’s experience with international treaties, such as ratifying the RtAF through the ICESCR, is shared by many LAC countries and has helped embed the RtAF into national institutional frameworks.^17^ These Spanish-speaking countries (and Portuguese-speaking in Brazil) have an opportunity to align their political, climate, social, and economic agendas within a comprehensive framework law on the RtAF.^15,17^ Within this regional context, parliamentary groups, such as the FPH, play a critical role in translating human rights commitments into durable legal frameworks, stable budgets, and long-term public policies.^17^

## Conclusion

 The LGAAS positions Mexico among a small number of countries adopting a rights-based framework to operationalize the RtAF from constitutional recognition into actionable food policy. This study contributes three main things: informant insights into the conditions under which the RtAF was operationalized through the LGAAS, nuances involved in the formulation process, and the way the RtAF was perceived in the law. The progressive realization of the RtAF through Mexico’s LGAAS now depends on effective law implementation – itself dependent on navigating competing actor interests and ongoing tensions that continue to affect the operationalization of the LGAAS.

## Acknowledgements

 This manuscript is based on a Master’s thesis completed as part of the Masters in Nutrition for Global Health from the London School of Hygiene and Tropical Medicine (LSHTM).

## Disclosure of artificial intelligence (AI) use

 The authors used AI tools (TurboScribe; ChatGPT) to translate the interview guides from Spanish to English, and for editorial assistance during manuscript preparation. All scientific content, interpretations, and conclusions were reviewed and approved by the authors.

## Ethical issues

 This study was approved by the Ethics Committee of LSHTM (Ethics Ref: 30051). Institutional ethical approval was obtained prior to beginning the interviews.

## Conflicts of interest

 Authors declare that they have no conflicts of interest.

## Disclaimer

 The lead author had an affiliation with LSHTM while conducting the research, but is not employed by the institution, and conducted the writing of the manuscript independently of this affiliation.

## References

[1] UN Economic and Social Council, General Comment No. 12: The Right to Adequate Food (Art. 11 of the Covenant). UN Committee on Economic, Social and Cultural Rights (CESCR); May 12, 1999. https://www.refworld.org/legal/general/cescr/1999/en/87491.

[2] Saul B. The International Covenant on Economic, Social and Cultural Rights: Travaux Preparatoires. Oxford University Press; 2016.

[3] Food and Agriculture Organization of the United Nations (FAO). Realizing the right to adequate food to end hunger and malnutrition in all its forms. Rome, Italy: FAO; 2023.

[4] Bourke-Martignoni J. The right to food. Research Handbook on Economic, Social and Cultural Rights as Human Rights. Edward Elgar Publishing. 2020:137-158.

[5] Mechlem K (2004). Food Security and the Right to Food in the Discourse of the United Nations. European Law Journal.

[6] Ayala A, Meier BM (2017). A human rights approach to the health implications of food and nutrition insecurity. Public Health Rev.

[7] De Schutter O. From charity to entitlement: Implementing the right to food in Southern and Eastern Africa. United Nations; 2012.

[8] Sage C (2014). Food security, food sovereignty and the special rapporteur: Shaping food policy discourse through realising the right to food. Dialogues in Human Geography.

[9] Pol JLV, Schuftan C (2016). No right to food and nutrition in the SDGs: mistake or success?. BMJ Glob Health.

[10] Harris J, Gibbons S, Kaaba OB, Hrynick T, Stirton R (2022). A ‘right to nutrition’ in its social, legal, and political context: how international human rights translate to Zambian realities. J Hum Rights Pract.

[11] Anderson, A.Moyo, B., Walls, H. Contemporary policy developments for addressing food insecurity and malnutrition through the operationalisation of the right to food. Economic & Social Rights Review in Africa. 2025;5(33). https://dullahomarinstitute.org.za/socio-economic-rights/esr-review-archives/archives/esr-review-no-5-vol-33-of-2025/esr-review-5-of-2025.pdf.

[12] Riol KSC. The right to Food Guidelines, Democracy and Citizen Participation: Country Case Studies. Taylor & Francis; 2016.

[13] Deutsche Welthungerhilfe e. V.Right to food. Bonn: Welthungerhilfe; 2024. https://www.welthungerhilfe.org/fileadmin/pictures/publications/en/fact-sheets/topics/2024-policy-brief-right-to-food-en.pdf.

[14] Latin America and the Caribbean Parliament. Framework Law on the Right to Food and Food Sovereignty. Panama City: Food and Agriculture Organization of the United Nations (FAO) and the Mexican Agency of International Cooperation for Development (AMEXCID); 2018:44.

[15] Fanzo J, Cordes KY, Fox E, Bulman A. Tying the knot: an interdisciplinary approach to understanding the human right to adequate nutrition. Colum J Transnat’l L. 2018;57:62. https://scholarship.law.columbia.edu/sustainable_investment_staffpubs/51.

[16] Pol JLV. El enfoque legal contra el hambre: el derecho a la alimentación y las leyes de seguridad alimentaria [The legal focus against hunger: the right to food and food security laws]. 2010. https://www.academia.edu/download/38643513/Enfoque_Legal_contra_el_Hambre_-_JL_Vivero__Henry_Dunant.pdf.

[17] Parliamentary Front Against Hunger in Latin America and the Caribbean. Regional FAO Office for Latin America and the Caribbean. 2010. http://parlamentarioscontraelhambre.org/file/info/fph-ialcsh.en.pdf.

[18] Government of Mexico. Iniciativa con proyecto de Decreto por el que se Expide la Ley General de Alimentación Sostenible [Draft decree issuing the general law for adequate and sustainable food]. Mexico: Senate of the Republic; 2024.

[19] Acedo A. Mexican Constitution Now Recognizes Right to Food. Foreign Policy in Focus. 2011. https://fpif.org/mexican_constitution_now_recognizes_right_to_food/.

[20] Frente Parlamentario Contra el Hambre[Parliamentary Front Against Hunger]. Plan de trabajo 2019 [Workplan 2019]. https://parlamentarioscontraelhambre.org/wp-content/uploads/pdf/Plan-de-Trabajo-2019-FPH-Final.pdf.

[21] FAOLEX Database. Food and Agriculture Organization of the United Nations (FAO); 2025. https://www.fao.org/faolex/results/en/?search=adv#querystring=c2VhcmNoPXJpZ2h0IHRvIGZvb2QgJmlubWV0YTpzdWJqZWN0c2VsZWN0aW9uPUZEJnllYXJGcm9tPSZ5ZWFyVG89JmZhb2xleF9jb3VudHJ5PU1FWCZlbmRzdHJpbmc9MQ==.

[22] Pineda E, Hernández FM, Ortega-Avila AG, Jones A, Rivera JA (2025). Mexico’s bold new law on adequate and sustainable nutrition. Lancet.

[23] Berlan D, Buse K, Shiffman J, Tanaka S (2014). The bit in the middle: a synthesis of global health literature on policy formulation and adoption. Health Policy Plan.

[24] Walt G, Gilson L (1994). Reforming the health sector in developing countries: the central role of policy analysis. Health Policy Plan.

[25] O’Brien GL, Sinnott SJ, Walshe V, Mulcahy M, Byrne S (2020). Health policy triangle framework: Narrative review of the recent literature. Health Policy Open.

[26] Buse K, Mays N, Colombini M, Fraser A, Khan M, Walls H. Making Health Policy, 3e. McGraw Hill; 2023.

[27] Food and Agriculture Organization of the United Nations (FAO). Transforming food and agriculture through a system approach. Rome: FAO; 2025. doi: 10.4060/cd6071en.

[28] Hennink M, Kaiser BN (2022). Sample sizes for saturation in qualitative research: A systematic review of empirical tests. Soc Sci Med.

[29] Carter N, Bryant-Lukosius D, DiCenso A, Blythe J, Neville AJ (2014). The use of triangulation in qualitative research. Oncol Nurs Forum.

[30] Jenkins-Smith HC, Sabatier PA. The study of public policy processes. The Nation’s Health. 1993:135-142.

[31] Haynes A, Loblay V (2024). Rethinking barriers and enablers in qualitative health research: limitations, alternatives, and enhancements. Qual Health Res.

[32] Baker P, Hawkes C, Wingrove K (2018). What drives political commitment for nutrition? A review and framework synthesis to inform the United Nations Decade of Action on Nutrition. BMJ Glob Health.

[33] Sing F, Carriedo A, Mackay S, Tenbensel T, Swinburn B (2023). Barriers and enablers in designing regulations to restrict the exposure of children to unhealthy food and beverage marketing. Front Polit Sci.

[34] Keeley J, Scoones I (2000). Knowledge, power and politics: the environmental policy-making process in Ethiopia. J Mod Afr Stud.

[35] Pérez-Ferrer C, Lock K, Rivera JA (2010). Learning from international policies on trans fatty acids to reduce cardiovascular disease in low-and middle-income countries, using Mexico as a case study. Health Policy Plan.

[36] Cervantes G, Thow AM, Gómez-Oliver L, Durán-Arenas L, Pérez-Ferrer C (2022). What opportunities exist for making the food supply nutrition friendly? A policy space analysis in Mexico. Int J Health Policy Manag.

[37] Rivera JA, Colchero MA, Pérez-Ferrer C, Barquera S (2024). Perspective: Mexico’s experience in building a toolkit for obesity and noncommunicable diseases prevention. Adv Nutr.

[38] Carriedo A, Lock K, Hawkins B (2020). Policy process and non-state actors’ influence on the 2014 Mexican soda tax. Health Policy Plan.

[39] MacKillop E, Downe J (2023). What counts as evidence for policy? An analysis of policy actors’ perceptions. Public Adm Rev.

[40] Moyo BH, Thow AMT (2020). Fulfilling the right to food for South Africa: justice, security, sovereignty and the politics of malnutrition. World Nutrition.

[41] Thow AM, Greenberg S, Hara M, Friel S, duToit A, Sanders D (2018). Improving policy coherence for food security and nutrition in South Africa: a qualitative policy analysis. Food Security.

[42] Villiers C, Farooq MB, Molinari M (2022). Qualitative research interviews using online video technology–challenges and opportunities. Meditari Accountancy Research.

